# Editorial: Culture, Self, and Autonomy

**DOI:** 10.3389/fpsyg.2020.00736

**Published:** 2020-04-28

**Authors:** Miriam Sang-Ah Park, Valery Chirkov

**Affiliations:** ^1^Jeffrey Cheah School of Medicine & Health Sciences, Monash University Malaysia, Subang Jaya, Malaysia; ^2^Department of Psychology, Nottingham Trent University, Nottingham, United Kingdom; ^3^Department of Psychology, University of Saskatchewan, Saskatoon, SK, Canada

**Keywords:** culture, autonomy, self, context, sociocultural models, relatedness, cross-cultural psychology

In our special topic *Culture, Self and Autonomy* we have examined the complex issues relating to how self and autonomy are explored, construed, and experienced by different subjects and across cultural contexts. The notion of the self stands at the center of the discussion on psychological autonomy, defined as a system of processes, including self-determination, self-regulation, and self-direction (e.g., Beck, 1997; Beck and Beck-Gernsheim, 2002; Ryan and Deci, 2017). Culture plays a key role in determining the basis of potentiality for autonomy, as it sets boundaries for the appropriate level of autonomy for individuals within a society (Chirkov, 2017). One of the primary dimensions of this topic was the development of the autonomous self in children and cultural differences in this developmental dynamic. Another important dimension has been the conditions for autonomy functioning in adolescents and young adults. The third dimension reflected in the submitted articles was an analysis of the macro-contexts and broad existential concerns as the background for autonomous functioning. The submissions to our special topic have been clustered along these three primary dimensions of our inquiry.

## Developmental Aspects of the Self and Autonomy Advancement

The work of Corapci et al. has contributed to such an understanding, linking young, educated Turkish mothers' self-construals to sensitive parenting. Their work has examined the role of autonomous-relational self-construal (Kagitçibaşi, 2007) in these mothers' parenting practice, highlighting how social change and the ensuing impact of education and changing socioeconomic status of the mothers have resulted in their self-views as well as how they have reared their children. These Turkish mothers have continued to value relatedness of the self while emphasizing the importance of autonomy. Such research evidence suggests that the cultural context continues to shape how one perceives the self and autonomy.

Komolova and Lipnitsky's interview-based study of mothers from the former Soviet Union has revealed that these mothers must balance their authoritarian attitudes rooted in their cultural background with more autonomy-supportive values and practices relevant to the North American context. The authors have suggested that this cross-cultural adjustment constituted the pivotal point of parental acculturation. Benga et al. examined children's self-regulation skills and related emotions in conjunction with their mothers' self-construals and socialization strategies. Within the limitations of a correlational study, this investigation demonstrated that the way children master their emotion and self-regulation strongly depends on their mothers' structure of the self (Independent vs. Interdependent) and their corresponding strategies for interacting with their children in different tasks.

## Autonomy Promotion in Adolescents and Young Adults

The work of Park et al. has compared various self-foci and explored the differences in how these selves manifest in the East Asian countries of South Korea and Japan. Their research has focused on the other-focused relational self, taking us away from a sole focus on autonomy of the self, emphasizing instead the relational/interdependent dimensions of the self-directed toward close others. The unique contribution of their research is that they found this other-focused relational self to be closely linked to self-esteem and well-being in Asians, which may present an alternative picture to the Western core beliefs and emphasis on autonomy and positive psychological outcomes. Soenens et al. focused on Asian adolescents' interpretations of parental practices. Their work found that the South Korean adolescents they studied held a relatively benign view of parental practices that can be seen as autonomy suppressive. The findings once again suggest that there are potential differences in how autonomy may be viewed or valued across cultures.

## Broad Contexts of the Autonomous Functioning

Robertson's conceptual article adds weight to our claim, as he has described the process of self-definition as a reflexive project that happens alongside and as the result of one's adaptation to his/her cultural and environmental experience. In such a sense, culture as a context is integral to one's construction of the self and autonomy. Robertson analysis has also taken us to the macro-level societal difference in ideologies that justify power relationships as what then influences how selves are experienced and managed in different cultures.

Grant has empirically investigated, using Google Ngram Viewer, a shift toward striving for purposeful life and collectivistic values in the millennial generation. The global shift toward collectivistic values, the author has found, is an interesting one, which gives further support to Kagitçibaşi (2007) in that the emphasis on relatedness and interdependence may be more prevalent across cultures than was previously thought to be the case.

McNamara and Reicher, the last published article on our special topic, have presented a somewhat different definition for autonomy in their study exploring airport surveillance experience, self, and autonomy. They have defined it as “the freedom to determine the self-one acts upon and to act on that self.” In such a view, the self is “variable” in that it changes as the result of a context one experiences. Their analysis has also pointed to the role of the other/perceiver and especially the authority in the way the self is experienced and how autonomy may be inhibited if the context does not allow one to experience the self as he/she wishes.

## Suggestions and Plans for Future Research

One of the main problems we wish to address in the future is the dialectical relations between cultures and people's ability toward autonomous agency and self-determination. The dilemma here is either that people are cultural beings determined and guided by cultural norms and prescriptions or that humans are autonomous agents who can go against cultural prescriptions, change the existent ones, or even create new ones. If we accept that humans are both cultural beings and are capable of autonomy and self-determination, then the question remains as to how cultural prescriptions and person's autonomy coexist and interact with each other. Ultimately, this concern boils down to the fundamental problem of structure and agency but with an inclination to address it at the psychological level. We wish to highlight the importance of examining the human self and its co-construction in different sociocultural environments.

We have also planned to place at the center of our upcoming investigations the concept of sociocultural models (Chirkov, 2016; Chirkov, in press) that shape people's selves and prescribe the forms of perceiving of and acting in the world. Of our focus here has thus ben interactions and interpersonal relationships with the members of the family (e.g., Allan, 2001; Park, 2015) and the closest circles of significant others as well as the broader social institutions and communities (Chua et al., 2019). Along the line of searching for such mechanisms, it will also be interesting to address the processes of culture learning and internalization, assimilation of cultural models, accommodation of cognitive representations of these models to ever-changing sociocultural realities, and development and evolution of one's intentional and intersubjective nature of sociocultural reality.

## Author Contributions

MP and VC edited the special topic, and have written the editorial together.

## Conflict of Interest

The authors declare that the research was conducted in the absence of any commercial or financial relationships that could be construed as a potential conflict of interest.
